# Multi-target bioactivity of summer quinones production in the Persian Gulf burrowing black-type sea urchin

**DOI:** 10.1016/j.heliyon.2022.e09044

**Published:** 2022-03-04

**Authors:** Soolmaz Soleimani, Sakineh Mashjoor, Morteza Yousefzadi, Manish Kumar

**Affiliations:** a Department of Marine Biology, Faculty of Marine Science and Technology, University of Hormozgan, Bandar Abbas, Iran; b Marine Pharmaceutical Science Research Center, Ahvaz Jundishapur University of Medical Sciences, Ahvaz, Iran; c Department of Biology, Faculty of Science, University of Qom, Qom, Iran; d Department of Pharmaceutical Engineering and Technology, Indian Institute of Technology, (BHU), Varanasi, India

**Keywords:** Spinochromes class, Echinochrome, Bioactive drug, Secondary metabolites, Persian Gulf

## Abstract

After harvesting the sea urchin gonads for Japanese food “uni” echinoculture systems, the remaining shells and spines are considered waste. However, the material of shells and spines is thought to be rich in natural bioactive molecules. The current study used liquid chromatography–electrospray mass spectrometry to extract summer quinones pigment present in spines and shells of the burrowing sea urchin ‘black’ type *Echinometra mathaei* from the natural Qeshm Island echinoculture. Then, the biochemical, antioxidant, anti-inflammatory, antidiabetic, antibacterial, and cytotoxic activities of sea urchin quinones pigment were investigated. In terms of bioactivity, both shell and spine pigments demonstrated strong radical scavenging activity (antioxidant). The shell pigment exhibited maximum albumin denaturation inhibition (IC_50_ = 9.62 μg/ml) (anti-inflammatory), as well as α-amylase inhibition (92.28 percent 4.77) (antidiabetic). Pigments were discovered to have a low antibacterial effect against positive gramme bacteria, as well as low cytotoxic and embryotoxic effects when compared to *Artemia salina* and zebrafish (*Danio rerio*). For identification and quantification of pigment extracts, both the photodiode array detector and LC-ESI-MS were used. Spinochrome A, B, and C, as well as echinochrome A, were identified as bioactive quinonoid pigments. This chemical defence is discussed in relation to its algal diet and environmental conditions. In conclusion, the isolated pigments obtained from the shell and spines of *E. mathaei* sea urchins found to have potent bio-activity and can be used for various biomedical and pharmaceutical applications.

## 1 Introduction

Marine biodiversity suggests a significant opportunity for the discovery of novel sources of bioactive natural compounds (Amarowicz et al., 2012). Sea urchins (Echinoderms/benthic marine invertebrates) have served as model organisms for scientific research for more than a century. The diet of sea urchins plays important role in controlling the community composition and structure of shallow subtidal macroalgae (Harrold and Reed, 1985). In many regions, intensive seaweed grazing and also feed of detritus, microalgae, and small invertebrates were used (Vadas Sr et al., 2000). A recent study highlighted that the sea urchins bioactive compounds are highly influenced by their diet (Kabeya et al., 2017; Sartori and Gaion, 2016).

Sea urchin roe (eggs) is considered as the expensive traditional delicacy “uni” in Japanese cooking (https://favy-jp.com/topics/2269) (Kuwahara et al., 2010) and fermented crop (Amarowicz et al., 2012). However, the remaining shells and spines are discarded as waste after harvesting the edible portions (Kuwahara et al., 2010). These shells and spines materials are rich in natural bioactive molecules, principally quinonoid pigments (spinochromes class) (Kuwahara et al., 2009; Hou et al., 2018). Spinochromes compounds are polyhydroxylated derivatives of either juglone (5-hydroxy-1,4-naphthoquinone) or naphthazarin (5,8-dihydroxy-1,4-naphthoquinone) with different functional groups (e.g., amino, ethyl, methoxyl, and acetyl groups) (Hou et al., 2018). Polyhydroxylated naphthoquinone (PHNQ) pigments from sea urchin have been shown to have pharmaceutical applications with a low toxicity profile (Sayed et al., 2018; Shikov et al., 2018). According to its primary sources, marine echinochrome contains PHNQs pigments that could be echinochromes or spinochromes (Hou et al., 2018). Based on their phenolic hydroxyl groups, spinochromes and Echinochrome A from sea urchin shells have been reported to have a variety of biological activities, including bactericidal, antialgal, antiulcerogenic, antioxidant, anticancer, hypolipidemic activity, and innate immune capacity (Soleimani et al., 2021; Dyshlovoy et al., 2020; Luparello et al., 2020; Fahmy et al., 2019; Coates et al., 2018; Sayed et al., 2018; Liu et al., 2018; Jiao et al., 2015; Zhou et al., 2011; Kuwahara et al., 2010).

Antioxidant scavenging activity is important in the prevention of many degenerative pathologies, including Alzheimer's disease, liver and cardiovascular disease, diabetes, Parkinson's disease, inflammation, arthritis, autoimmune and neurodegenerative disorders, and atherosclerosis (Uttara et al., 2009; Yun-Zhong et al., 2002). Given the safety limits of synthetic antioxidants like butylated hydroxytoluene (BHT) and/or butylated hydroxyanisole (BHA) in lipid-containing foods (Pokorný, 2007), there is growing interest in natural antioxidant sources for dietary and/or environmentally friendly use. Furthermore, despite the fact that PHNQs pigments have demonstrated significant bioactivity, differences in composition make comparison of pigment efficacy with known drugs difficult. The current research aimed to investigate the following: (1) extract and summer bioactivity profiling of the quinonoid pigments in the spines and shells of the Persian Gulf ‘black’ type sea urchin *Echinometra mathaei*, (2) attempt to separate pigments and their quantitative and qualitative characterization, (3) introduce PHNQs pigments from natural environments and/or echinoculture systems as a new source of natural antioxidant, antidiabetic, antibacterial, antiinflammatory, and cytotoxicity compound for medicinal use.

## 2 Methods

### 2.1 Collection, extraction, and processing of pigments

In the Persian Gulf, Qeshm Island shallow subtidal zone (26^◦^55′N, 56^◦^16′E), is a vast natural echinoculture. A total of 25 black type sea urchin *E. mathaei* with a mean weight of 51.15 ± 2.83 g was captured in July 2015 from these regions (according to the tide time table: www.tides4fishing.com, www.tide-forecast.com) of Qeshm Island in the Persian Gulf. The specimen recognition was carried out by using taxonomic keys (Price, 1986). For rapid killing and anaesthetization, sea urchins were sacrificed in chilled water (AVMA, 2013). The shells and spines collected were immediately rinsed in the laboratory and the pigments were extracted according to Kuwahara et al. (2009) method. In brief, the sea urchin shells and spines were collected, washed with tap water, freeze-dried, and then powdered. Approximately 10 g of the shell and spine powder was dissolved in 100 ml of 6 M HCl at a solid to liquid ratio of 1:10 (w/v) in the dark at 20 °C. The shells and spine pigments were extracted with 100 ml of diethyl ether. The ether layer was washed with 5 % NaCl, until the acid was removed. The ether extract containing the pigments was dried, and the solvent was evaporated (Amarowicz et al., 2012). Then the weight of pigments was calculated, and the PHNQ pigments were redissolved in DMSO and placed in freezer at -20 °C in the dark. In all experiments, each sample was assayed in technical or biological triplicate and deionized water was used for suspension and dilutions.

### 2.2 HPLC-MS analysis

To accurately separate and check the major *E. mathaei* quinonoid pigments in shells and spines, the optimized liquid chromatography coupled with ion trap mass spectrometry (LC-MS) was executed using the standard protocol (RSC Analytical Methods Committee, 2013). Prepared samples (20 μL) were analyzed on an LCQ-DECA system, comprising an Agilent LC 1200 series liquid chromatography coupled to a Thermo Finnigan mass spectrometer ion trap (Thermo Scientific, Hemel Hampstead, UK). HPLC condition including C18 column (250 9 4.6 mm, 5 lm), a solvent system delivered at a flow rate of 0.5 mL/min and consisted of a mixture of solvent (A) formic acid/water (0.1:100, v/v) and solvent (B) MeOH/acetonitrile (5: 9, v/v) were applied, so that the mobile phase was 50 % mixture of solvents (A) and (B) in an isocratic elution. ESI was set in a negative mode in a condition including sheath gas: 60 mL min-1, auxiliary gas: 20 mL min-1, spray voltage: 4.5 kV, capillary temperature: 200 C, capillary voltage: 46 kV, and tube lens: -60 kV). The Xcalibur 2.0 SR2 software (copyright Thermo Electron Corporation, 1998–2006) was used. To HPLC method validation, the linearity of the method was established by using stock solutions of standard samples of Echinochrome A and Spinochrome A-E in methanol at different levels of 100–1300 ng/mL. Calibration curves were plotted by drawing the height of the negative peak vs concentration (Pozharitskaya et al., 2013a, b). The limits of quantification (LOQ) and detection (LOD) of standard samples were measured as levels at which the signal-to-noise ratio (SNR) is <10 and <3, respectively.

### 2.3 Pigments quantity determination

After HPLC-MS analysis, the absorption spectra of verified pigments were identified by spectrophotometer (Kuwahara et al., 2010). The quantity of pigment was measured based on molar extinction coefficient (ε) that refers to echinochrome A (ε = 7413 at 490 nm), spinochrome A (ε = 3311 at 520 nm), spinochrome B (ε = 4898 at 480 nm), and spinochrome C (ε = 5888 at 463 nm) (Kuwahara et al., 2010). Like this, the naphthoquinone pigments chemical structures in shells, and spines of sea urchin were recognized (Anderson et al., 1969). The quantity of pigment was calculated as follows (Eq. 1):

(1) A = εcl

where A is the solution absorbance, ε is the molar absorptivity (mol L^−1^ cm^−1^), c is the concentration of the pigments sample (mol L^−1^) and l is the path length of light in 1 cm.

### 2.4 Antioxidant assay

#### 2.4.1 DPPH radical scavenging activity

Free radical scavenging (FRS) activity of different concentrations of the pigments (12.5, 25, 50, 100, 200, 400, and 800 μg/ml) against DPPH (1, 1-diphenyl 2-picrylhydrazyl) (Sigma, St. Louis, MO) was measured by using Duan et al. (2006) method. Briefly, 0.1 mL of pigment extracts and 0.1 mL of DPPH solution (0.5 mM in methanol) were mixed and incubated at dark (30 min). Then the absorbance of the sample was assessed at 490 nm. Then, the DPPH radical inhibition by the pigments extract was calculated (Eq. 2):

(2) DPPH scavenging activity (%) = 100% × [1−(A_s_−A_0_/A)]

where, A_s_ is the absorbance of the reaction solution, A_0_ of the blank, and A of the control. The analyses were performed in triplicate and BHT as a positive control.

#### 2.4.2 Total antioxidant capacity (TAC)

Total antioxidant activity of the PHNQs pigment extract was measured according to the method of Mitsuda et al. (1996). Briefly, 7.45 mL of sulphuric acid (0.6 M), 0.99 g of sulphate sodium, and 1.23 g of ammonium molybdate were mixed together in 250 mL with distilled water as a TAC reagent. Then 0.1 mL of the pigments extract with different concentrations (50, 100, 250, 500 μg/mL) was dissolved in 1 mL of TAC solution. After 15-min incubation in the RT, the absorbance was read at 695 nm. Ascorbic acid (vitamin C, at 50–1000 μg/mL) was used as a positive control.

### 2.5 Antiinflammatory assay

The ability of urchin pigments to blockage thermal and hypotonic protein denaturation was considered as their antiinflammatory properties. To evaluate the potential antiinflammatory effects of pigment extract solution at different levels (1.25, 2.5, 5, 10, 20 μg/ml), the method of Aguilar-Toalá et al. (2017) and Mizushima and Kobayashi (1968) was used. In brief, the reaction mixture inclouded of 0.45 mL of the bovine serum albumin (0.1% BSA, pH = 6.3) and 0.05 mL of pigment extract (in DMSO). The solution was sequently incubated at 37 °C and then at 57 °C (20 min). After cooling the solution, the turbidity was measured at 660 nm and then the percentage inhibition values for the protein denaturation was measured (Eq. 3):

(3) % inhibition of protein denaturation = 100% × (A−A_s_)/A

where A is the control absorbance and A_s_ is the reaction solution absorbance. The analyses were performed in triplicate and Aspirin (Acetylsalicylic acid) as the positive control.

### 2.6 α-Amylase inhibition assay

The method of Ademiluyi et al. (2014) with minor modifications was adapted for carrying out α-amylase inhibition assay which involved in colorimetric reaction by 3, 5-dinitrosalicylic acid (DNSA) and starch. In this colorimetric assay, the used substrate and reagent were starch and DNSA, respectively. To calculate the quantity of maltose, the standard curve was drawn. The α-amylase solution (4 unit/mL) was prepared by mixing 0.001 g of porcine pancreatic α-amylase (EC 3.2.1.1) in 20 mM sodium phosphate buffer (pH = 6.9), which containing 6.7 mM sodium chloride. The starch solution and pigment extracts (in DMSO) were integrated. The DNSAreagent possessed 20 mL of 96 mM 3, 5-dinitro salicylic acid, 8 mL of 5.31 M potassium tartrate sodium in 2 M sodium hydroxide, and 12 mL of deionized water. In a tube, approximately 0.56 mL of the extracted-starch solution and 0.04 mL enzyme were combined and incubated at 37 °C (15 min). Next, added 0.6 mL of the DNSA, and for 15 min, the test tube was incubated at 85 °C. Then, by using a spectrophotometer, the absorbance of the samples was determined at 540nm. Acarbose was used as a positive control. The percentage of inhibition of α-amylase was determined (in triplicate) by the following (Eq. 4]:

(4) $${\text{I}}_{\text{α}-\text{amylase}}\%=\left(\left(\text{Δ}{\text{A}}_{\text{cotrol}}-\text{Δ}{\text{A}}_{\text{sample}}\right)/\text{Δ}{\text{A}}_{\text{control}}\right)\times 100$$

$$\text{Δ}{\text{A}}_{\text{control}}={\text{A}}_{\text{test}}-{\text{A}}_{\text{blank}}$$

$$\text{Δ}{\text{A}}_{\text{sample}}={\text{A}}_{\text{test}}-{\text{A}}_{\text{blank}}$$

### 2.7 Antimicrobial assay

Seven strains of pathogenic Gram-positive and negative bacteria were used in the antimicrobial activity assay: *Bacillus subtilis* (ATCC 465), *B. pumilus* (PTCC 1274), *Staphylococcus aureus* (ATCC 25923), *Escherichia coli* (ATCC 25922), *Serratia marcescens* (ATCC 13880), *Vibrio alginolyticus* (ATCC 17749) and *Vibrio logei* (ATCC 15382). Based on the method of Mashjoor et al. (2016), minimum inhibitory concentration (MIC) values for pigment extract (1000, 500, 250, or 125 μg/ml) were determined using a broth microdilution assay (in triplicate). For each microorganism, an overnight Mueller–Hinton Broth culture was prepared and adjusted with 0.5 McFarland turbidity standards. Approximately 0.1 ml of a suspension test was spread over Mueller–Hinton Agar plates. On the agar plates, filter paper discs (6 mm diameter) that were impregnated with 10 μl of pigment extract were placed. Disks with ampicillin (at 10 μg/disc) were used as controls and the plates were incubated at 37 °C (overnight). Finally, diameters (mm) of each sample inhibition zones were measured.

### 2.8 Cytotoxicity and embryotoxicity assay

#### 2.8.1 Brine shrimp lethality assay (BSA)

By using Mashjoor and Yousefzadi (2019) method with brine shrimp, *Artemia salina,* the cytotoxicity assay with shell and spine pigment concentrations between 125-1000 μg/ml was performed in triplicate. In brief, *A. salina* cysts (Oji Art Industries, Japan) were hatched in a 3.5% NaCl solution. After 48 h, the nauplii were collected and transferred to 24-well plates containing seawater (pH = 8.8, Salinity = 35 ‰; 2 mL/10 nauplii). Different levels of pigment extract (125, 250, 500, and 1000 mg/mL) were prepared in DMSO and mixed with vials solution. After 24 h, the number of lived and deaded nauplii was counted at each dose and control (seawater with DMSO). By following the formulae, the percentage mortality was determined (Eq. 5):

(5) %Mortality ​= ​Number ​of ​dead ​nauplii/Initial ​number ​of ​live ​nauplii

By plotting the results as a log of % mortality vs log concentration, the median lethal dose (LC_50_) was calculated.

#### 2.8.2 Zebrafish embryo acute toxicity test (ZFET)

For the ZFET test, adult wild-type zebrafish (*Danio rerio*) were obtained from Gorgan Zebrafish Aquaculture Core Facility, Iran. The fish embryo acute toxicity of the test (Lammer et al., 2009) was performed according to OECD TG 236 (OECD, 2013). In brief, freshly zebrafish eggs (<1 h post-fertilisation) were collected with a pipette and individually transferred (10 eggs) to 24-well plates with 2 ml of test solution per well. Subsequently, different concentrations (10, 50, 100, 200, 500 μg/mL) of pigment extract were prepared in DMSO and mixed with vials solution (n = 6). Well plates were placed in an incubator at 27.0 ± 1.0 °C under a 10/14-h dark/light regime. All development status of the zebrafish embryos were observed under stereomicroscope (Olympus BX40, USA) and documented at 12, 24, 36, and 48 h. According to the OECD TG 236, coagulated embryos (milky white eggs), the absence of somite formation, non-detachment of the tail and lack of heartbeat was recorded as lethal or sublethal endpoints. LC and/or effect concentrations (EC) were calculated by EPA Probit Analysis Program (Version 1.5).

### 2.9 Statistical analysis

All data were presented as means ± SD (n = 3). By using SPSS 19 and Excel 2013 software, all analyses were performed. To determine the significant differences between the means of various groups, a one-way analysis of variance (ANOVA) and Duncan's new multiple-range tests were used. p-values ≤ of 0.05 were considered significant.

## 3 Results and discussion

**Figure 1:**
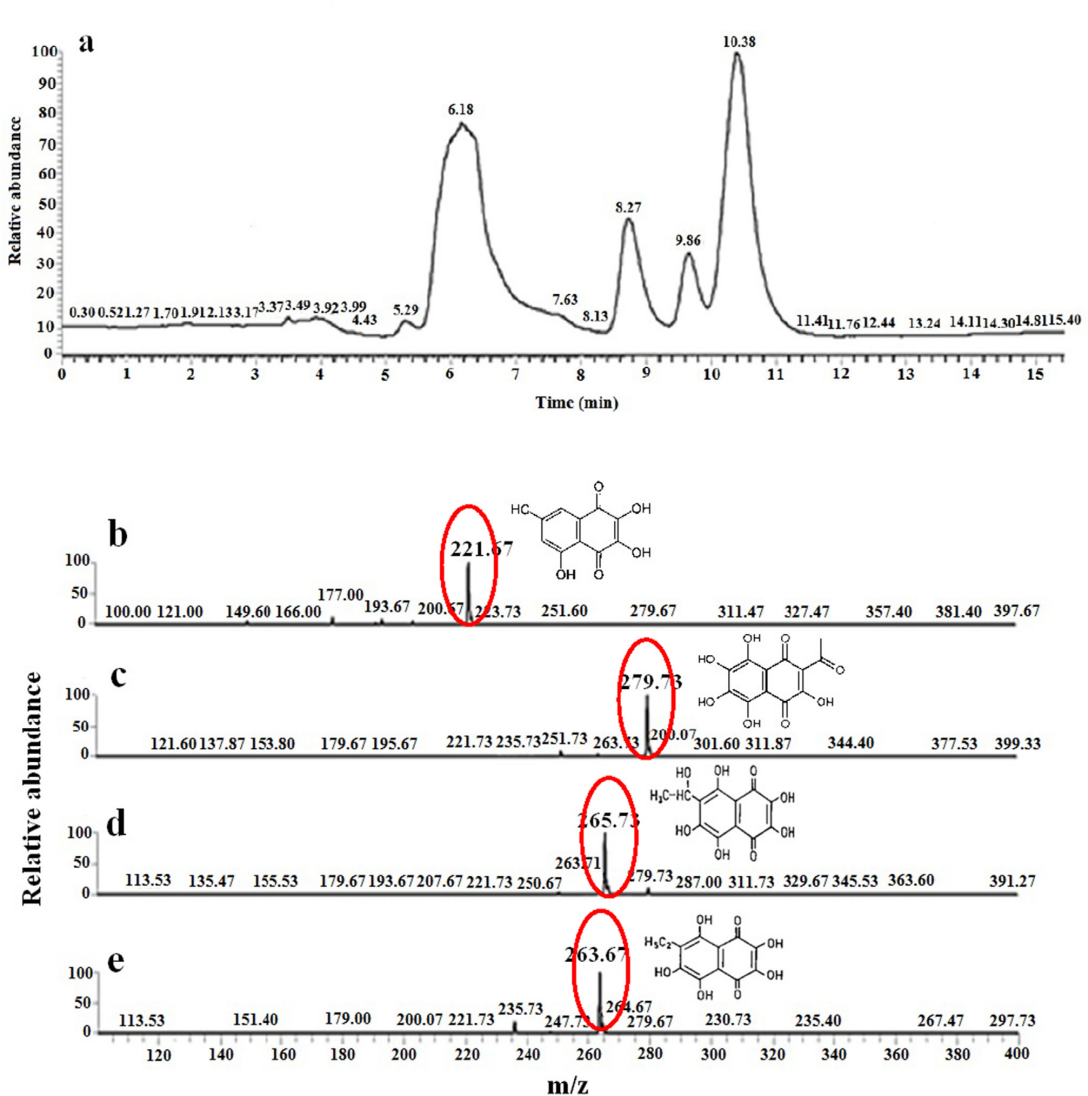
(A) Total Ion Current chromatogram (TIC, negative mode) of PHNQs pigment extracts samples studied during Liquid Chromatography Electrospray Ionization Tandem Mass Spectrometry; (B–E) Mass spectra; B, C, D, and E correspond to Spinochrome B and C, Echinochrome A, and Spinochrome A, respectively.

**Figure 2:**
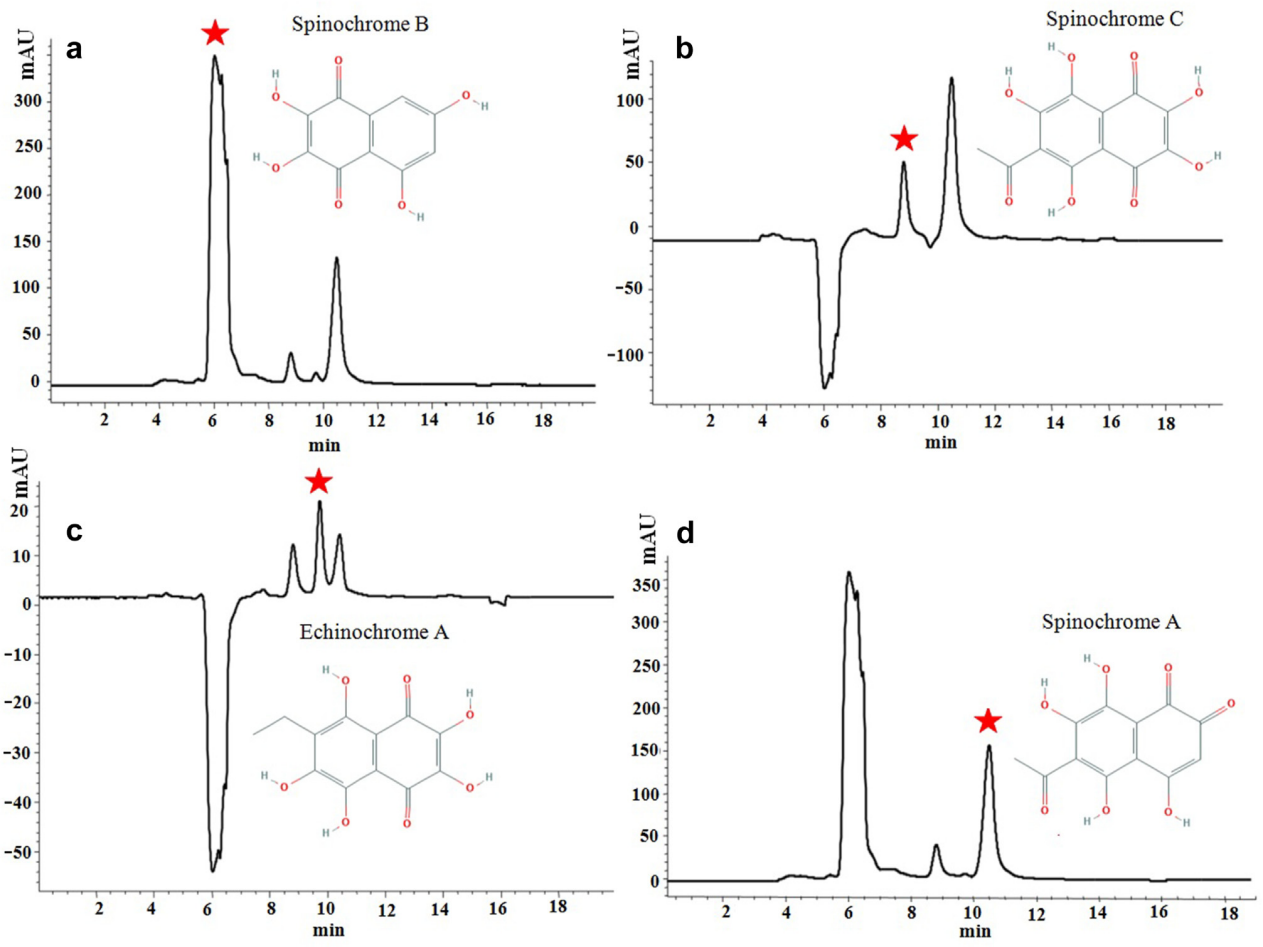
HPLC–DAD characterization of the *E. mathaei* PHNQ pigments (A–D).

Total *E. mathaei* pigment yield after extraction for the shell and spine is 24.0 mg and 27.9 mg per dry extract, respectively. These yields of PHNQ pigments are in agreement with those reported for *Evechinus chloroticus* shell and spines (21.0 mg and 7.9 mg dried material, respectively) (Hou et al., 2019), *Strongylocentrotus franciscanus* shells (12.1 mg dried material), and *S. droebachiensis* shells (16.3 mg dried material) (Amarowicz et al., 2012) by using the same solvent (diethyl ether). However, the pigment content variation in the literature reflected the difference in ecological niches and geographic distributions for each sea urchin species and different PHNQ extraction methods. For PHNQs pigment extracts, typical HPLC-DAD chromatograms were recorded at the most common of their λ_max_ (285, 317, 323, and 343 nm for Spinochrome C, A, B, and Echinochrome A, respectively). Since they are completely separate, their UV pattern and their different λ_max_ could confirm the presence of the PHNQs. Further verification was made using a liquid chromatography-electrospray mass spectrometry. In Figure 1 A–E the total ion current chromatogram of the pigments is illustrated. As shown in Figure 2 A–D, four peaks were obtained using HPLC-DAD. Since the mass spectrometer was operating in negative mode, all pigments appeared in pseudo molecular ions [M-H]^-^. As a result, ions at m/z 221, 279, 265, and 263 are pseudo molecular ions [M-H]^-^ of spinochrome B, C, echinochrome A, and spinochrome A, respectively (Figure 1 B–E). The percentages of identified main quinonoid pigments in 1 mL of *E. mathaei* shells and spines pigment extracts in the range of 2–5%. The liquid chromatography-mass spectroscopic (LC-MS) results confirm *E. mathaei* possess PHNQs pigments analogous to other sea urchins (Li et al., 2013). These spinochromes were identical to those found in the *S. nudus*, *S.intermedius*, *Glyptocidaris crenularis*, and *Anthocidaris crassispina* (Kuwahara et al., 2010; Li et al., 2013). As reported, *S. franciscanus* (Amarowicz et al., 2012) is red-brown and *S. droebachiensis* is green (Amarowicz et al., 2012; Shikov et al., 2011), but these species are purple. Therefore, various coloured sea urchins variety in the PHNQs compound has been identified (Amarowicz et al., 2012). However, the high concentration of spinochromes in black type *E. mathaei* suggested their significance in Echinoids' fitness (as a protective agent against microorganisms, UV radiation, and ROS) (Brasseur et al., 2018a). Although, the comparative amounts were diverse which is not expected given that *Psammechinus miliaris* (Powell et al., 2014). Previously, Vasileva et al. (2021) have described Echinochrome A as the predominant quinonoid pigments in the shells and spines from the following sea urchins: *Maretia planulata* (94%), *Diadema setosum* (92%), *Echinocardium cordatum* (up to 88%)), *D. savignyi* (83%), *Stomopneustes variolaris* (81%), and *Toxopneustes pileolus* (80%); as well as Spinochrome A, Spinochrome E, binaphthoquinone 11, spinochromes C, and Spinochrome D were present as the main compounds in *Phyllacanthus imperialis* (77%), and *T. gratilla* (67%), *Echinarachnius parma* (up to 52%), *Laganum decagonale* (up to 45%), and *E. parma* (up to 14%), respectively. In addition, the symbiotic interaction and host chemical recognition mechanisms presented by *E. mathaei,* spinochrome pigments have an essential role in the ecto commensal and metabolism of shrimps (Brasseur et al., 2018b). However, the distinct role of PHNQs pigments is not clear in the urchin shells and spines. PHNQs are accumulated only in the living dermis layer of the shell, which are mainly framed of the calcareous skeletal elements. Accordingly, the PHNQs may have a protective function (Powell et al., 2014) and will be locally willing at higher concentrations in relation to the external aqua-environment. Brasseur et al. (2018c) suggested that in the mapping of spinochromes of Madagascar sea urchin (*D. savignyi*, *T. gratilla* and *T. pileolus*), 11 different spinochromes were recognized including Spinochrome D–Iso 3, and Echinochrome A, having a body pattern similar to the *E. mathaei*. Alternatively, separate studies have demonstrated a correlation between sulphate group derivatives of spinochromes and changes in the bioactivities of marine natural products (Ortega et al., 2017; Zhang et al., 2011). Based on the varied seasonal allocation of energy and nutrients, ecological roles, and the life support of each sea urchin species, it is expected that these urchin secondary metabolites may differ substantially (Vasileva et al., 2021). The diets of sea urchin consist intensive an even mixture of macroalgal (Vadas, 1990) (e.g., the genus of *Ulva* (Chlorophyta (*Padina*, *Cystoseira*, *Sargassum*, and *Laminaria* (Phaeophyta (*Acanthophora, Palmaria* and *Gracilaria* (Rhodophyta (Westbrook et al., 2015; Vadas Sr et al., 2000; Hay et al., 1986). Though bioactivity assessment of Qeshm Island seaweeds from the Persian Gulf, Iran were previously reported for the majority of these algal species (Soleimani et al., 2018; Zarei Jeliani et al., 2018; Mashjoor et al., 2016; Mosaddegh et al., 2014; Namvar et al., 2014; Kokabi et al., 2013; Sadati et al., 2011), it seem that *E. mathaei* bioactivities from the Persian Gulf may be influenced by algal diet, macroalgae bloom, photoperiodic and temperature regimes and gametogenesis stages during summer (Shpigel et al., 2004; Vadas Sr et al., 2000).

### 3.1 Antioxidant activity

**Figure 3:**
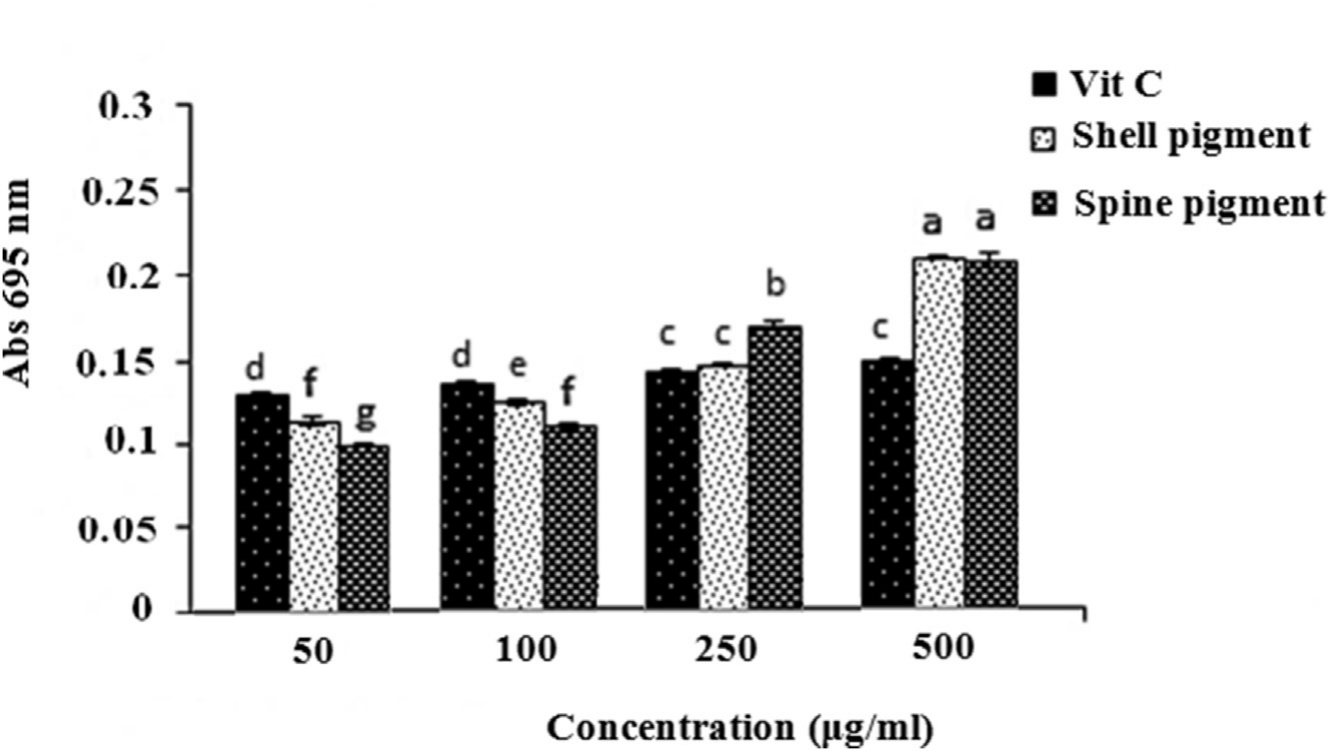
Total antioxidant capacity (TAC) of the shell and spine pigments isolated from *E. mathaei*.

The total antioxidant capacity of both shell and spine pigments possessed a substantial dose-dependent effect at a concentration range of 50–500 μg/ml. The results were displayed that the TAC capacity of the pigments higher than that of the well-known synthetic antioxidant, vitamin C at concentrations of >250 μg/ml (Figure 3). This antioxidant activity of pigments can be mainly linked to the bioactive compounds in the sea urchin PHNQ-metabolites. As reported by Vasileva et al. (2021) and Hou et al. (2020) the antioxidant effect of the natural PHNQs from shells and/or spines of sea urchin depend on their structural features such as hydroxylation, methylation, and the degree of sulfation which can act as a reactive oxygen species (ROS) inhibitor. Alternatively, Powell et al. (2014) exhibited that the antioxidant activity of sea urchin PHNQ-enriched pigment extracts can be mainly attributed to the range of sulphated or phosphorylated derivatives of spinochrome and echinochrome PHNQ components. However, in general, the antioxidant activity of the sulfated/phosphorylated derivatives was stronger than that of the natural PHNQ, the nature and extent of these modified PHNQ pigments in *E. mathaei* is not known and further work is required.

**Figure 4:**
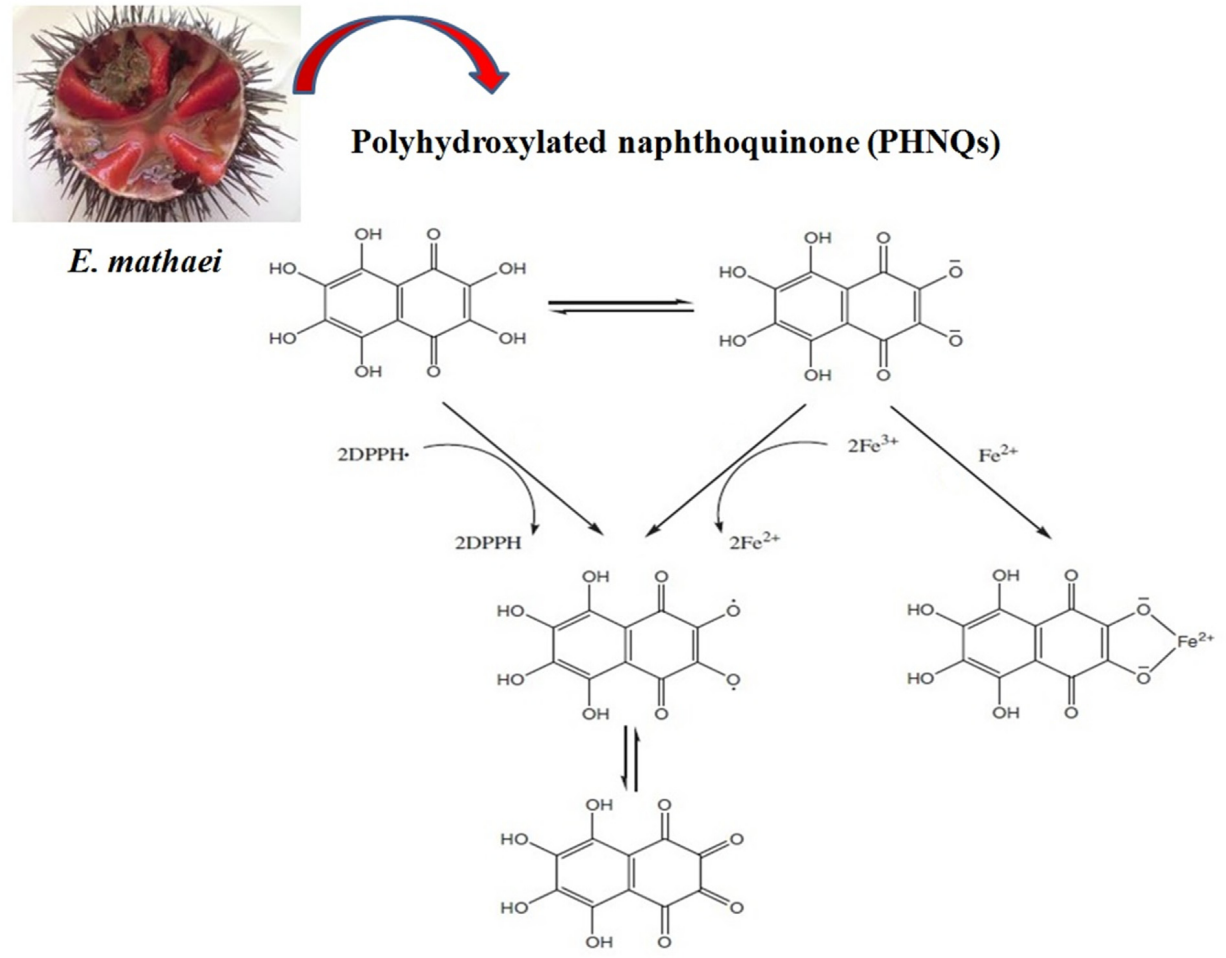
The antioxidant mechanisms of PHNQs pigments from *E. mathaei*.

The pigment of the black type *E. mathaei* shell shows almost potent DPPH radical scavenging activity compared to spine pigment which agreed with Zhou et al. (2011) and Pozharitskaya et al. (2013a, b). As shown in Table 1, both pigments from the shell and spine were effective in reducing radical DPPH to 1, 1-diphenyl 2-picrylhydrazyl. The radical scavenging activity of both shell and spine pigments incremented in a concentration-dependent manner when contrasted with BHT (Kuwahara et al., 2009). By losing two H atoms consecutively, PHNQs formed intermediate and final products of naphtha-semiquinone and naphtha tetraketone, respectively (Figure 4) (Lebedev et al., 2001). The iron-retaining strategy of echinoid innate immunity to suppress oxidizing radicals and microbial deterrent is well-modulated by dual immunocompetence functionality of echinochrome A to switch between oxidized and reduced forms (Coates et al., 2018; Li et al., 2013) (Figure 4). Echinochrome A released by primary immune cells of red spherule cells (RSC) is often recognized near damaged spines (*in vivo*) (Zapata-Vívenes et al., 2021; Hira et al., 2020; Coates et al., 2018). RSCs are responsible for the biogenesis of naphthoquinone compounds derived from spinochrome dimers/echinochrome and for the regulation of their bioactivity function (Hira et al., 2020). Sea urchin is also a violent seaweed grazer, specifically green and brown algae, and is therefore expected to bio-accumulate some of the antioxidant compounds (Amarowicz et al., 2012; Kuwahara et al., 2010; Mamelona et al., 2011; Vadas Sr et al., 2000). The results of the DPPH• scavenging assay of summer monitored seaweeds (such as *Padina pavonica*, *Colpomenia sinuosa*, *Cystoseira myrica, U. lactuca, U. linza, U. intestinalis Acanthophora muscoides*, *Chondrophycus papillosus, Sargassum swartzii,* and *S. angustifolium*) from the coastal waters of the Qeshm island, showed an antioxidant potential (IC_50_ 62–288 μg/ml; total phenol content (TPC, 27–36 mg GA/g DW; total flavonoid content (TFC,1.2–3.8) and obvious iron-chelating ability) (Soleimani et al., 2018; Zarei Jeliani et al., 2018; Kokabi et al., 2013; Sadati et al., 2011), and providing valuable minds for the expansion of sea urchin medicinal antioxidants. Recently, several natural phyto-biodynamic compounds such as polyunsaturated fatty acids, polysaccharides, phlorotannins, tocopherols, carotenoids, stypoldione, terpenes, and sterols have been identified in seaweeds to possess antioxidant potent and therapeutic value (Shahidi and Rahman, 2018).

**Table 1:** DPPH radical scavenging activity of pigments shells and spines extracts.

Samples	Concentrations (μg/ml)						
	12.5	25	50	100	200	400	800
BHT	3.47 ± 0.48^e^	4.49 ± 0.75^e^	24.56 ± 0.95^d^	47.68 ± 0.28^c^	53.73 ± 0.49^b^	56.88 ± 1.56^b^	92.89 ± 0.41^a^
Shell pigment	21.56 ± 0.28^e^	31.57 ± 0.66^d^	75.57 ± 0.59^c^	91.60 ± 0.99^b^	93.62 ± 1.05^b^	97.63 ± 0.97^a^	99.66 ± 0.80^a^
Spine pigment	39.50 ± 0.68^de^	41.52 ± 0.55^d^	47.52 ± 0.70^d^	57.53 ± 0.80^c^	64.58 ± 0.65^bc^	75.62 ± 0.77^b^	87.66 ± 0.70^a^

Values are mean ± SD (n = 3); DPPH values are expressed as μg/ml; Significant differences are indicated by different letters as determined by Duncan's Post-Hok multiple comparison (p < 0.05).

### 3.2 Antiinflammatory activity

The ability of the extract to suppress the albumin denaturation at different concentrations was also evaluated to determine the potential antiinflammatory effects (Table 2). The maximum inhibition (67.74 ± 1.35%) of shell pigment extracts was at 20 μg/ml and the minimum inhibition was at 1.25 μg/ml. In comparison, the PHNQs of natural origin isolated from spine pigment extract showed a minimum inhibition at 2.5 μg/ml (Table 2). Mechanistic features of sea urchin inflammatory programs are largely modulated by intracellular levels of Ca^2+^ that trigger echinochrome discharge from RSC (Coates et al., 2018). Besides, the antiinflammatory activities of shell polysaccharide of sea urchins, *Strongylocentrotus nudus*, *Glyptocidaris crenularis*, and *Anthocidaris crassispina* and antiallergic effects of shell PHNQs of green sea urchin, *S. droebachiensis* were also reported (Jiao et al., 2015; Pozharitskaya et al., 2013a, b). Similar to other antiinflammatory drugs such as salicylic acid, phenylbutazone, flufenamic acid, etc, PHNQs have also demonstrated a dose-related ability to cause thermally induced protein denaturation (Mizushima and Kobayashi, 1968). Viewed in conjunction with the reports of Kundakovic et al. (2004) naphthazarin derivatives could inhibit and modulate inflammatory reactions by up-regulation of pro-inflammatory cytokines and antiinflammatory cytokines. It may be because of high extract concentrations, responsible for a conformational change in the enzyme-compounds binding. In addition, the antiulcer healing effect of quinonoid pigment against gastric peptic ulcer-induced oxidative stress has been affirmed by a substantial change in the behavior of malondialdehyde (MDA), Superoxide dismutases (SOD), catalase (CAT), glutathione S-transferase (GST), Glutathione (GSH) content and nitric oxide (NO) level in rats (Hou et al., 2018).

**Table 2:** Inhibitory action of pigments shell and spine extracts on protein denaturation.

Test sample	Concentration (μg/ml)	% protein inhibition	IC_50_ (μg/ml)
Shell pigment	1.25	32.92 ± 1.24^e^	9.62
	2.5	36.30 ± 0.88^de^	
	5.0	45.63 ± 1.44^c^	
	10.0	50.62 ± 0.70^bc^	
	20.0	67.84 ± 1.35^a^	
Spine pigment	1.25	0.00 ± 0.00^h^	----
	2.5	0.00 ± 0.00^h^	
	5.0	4.64 ± 1.40^h^	
	10.0	16.10 ± 0.40^g^	
	20.0	22.72 ± 2.00^f^	
Aspirin	1.25	39.83 ± 1.30^d^	3.84
	2.5	47.90 ± 1.91^c^	
	5.0	54.11 ± 1.49^b^	
	10.0	47.71 ± 1.33^a^	
	20.0	71.21 ± 1.57^a^	

Values shown as means ± SD (n = 3); Significant differences indicated by different letters (p ≤ 0.05).

### 3.3 α-Amylase" inhibition

**Figure 5:**
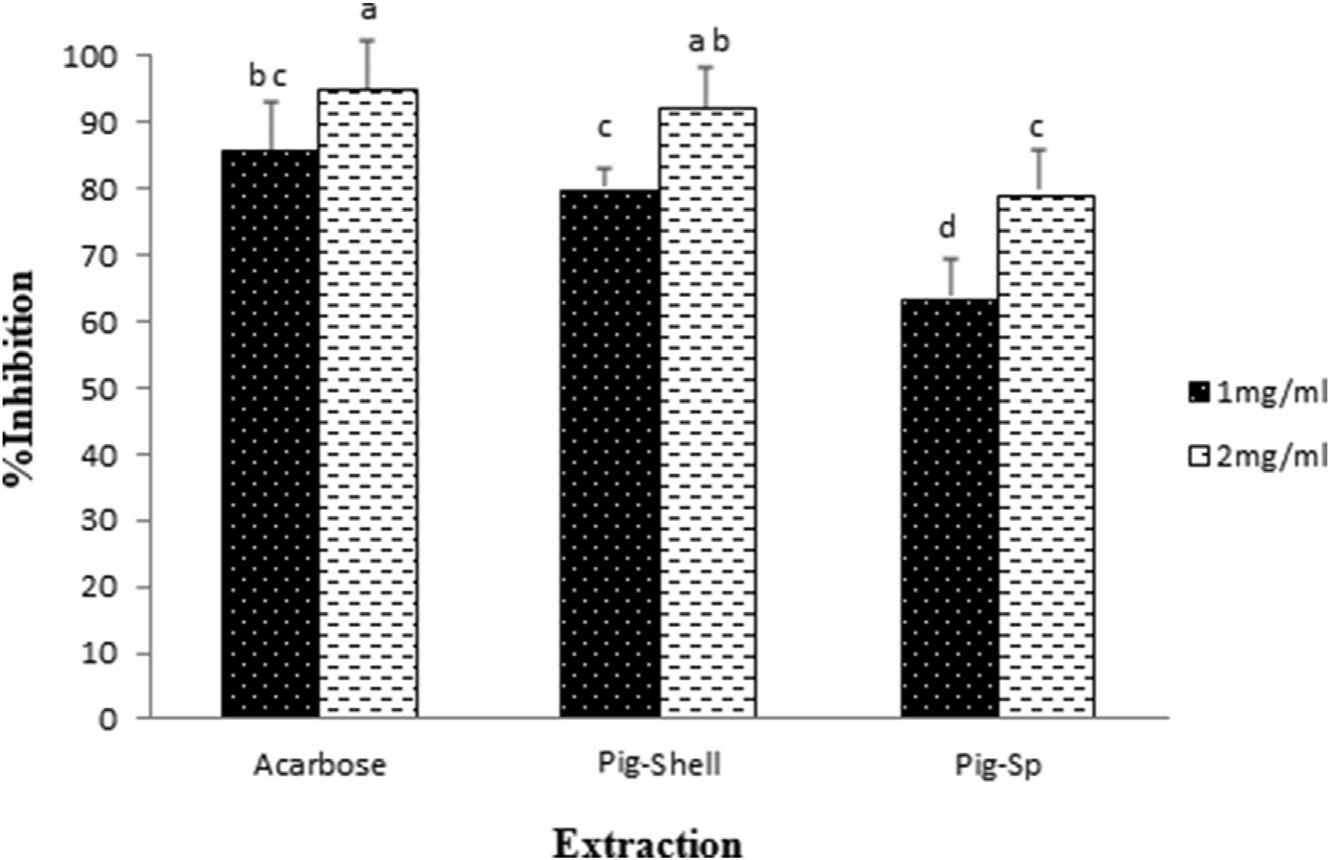
Percentage (%) inhibition of amylase pigment shell and spine against starch at pH 6.9. Significant differences are indicated by different letters (determined using Duncan's Post-Hok multiple comparison tests; p ≤ 0.05).

As observed in Figure 5, the highest α-Amylase" inhibition was found to be 92.28 ± 4.77% for shell pigment (at 2 mg/ml concentration) compared to acarbose as a positive control (95.03 ± 3.35% at 2 mg/ml).

The results showed that among the extracts analyzed, a desirable relationship was established between the inhibitory effect of α-amylase and antioxidant activity, especially for shell pigment. In this regard, Kovaleva et al. (2013) and Soliman et al. (2016) reported that the administration of sea urchins complex containing PHNQs pigments and minerals shells to the streptozotocin-nicotinamide-induced type II diabetic mouse model exhibited a decrease in blood glucose levels, oxidative stress parameters and improved musculoskeletal conditions and lipid and protein metabolism. However, recently sodium salt of echinochrome A, a quinonoid pigment of sea urchins (Histochrome) was proposed as a hypoglycemic agent in the treatment of diabetes, but still certain teratogenic risks were found in the pregnant Wistar Rats at a high dose of echinochrome (Mohamed et al., 2019). Therefore, it may be important to quantify the optimal dose of *E. matheai* quinonoid pigment in the current study based on the activation of the creatinine kinase enzyme by echinochrome in hypoglycemic conditions (Jevrić-Čaušević et al., 2006). In addition, Rohn et al. (2002) demonstrated that phenolic substances capable of forming quinones (i.e., chlorogenic acid, caffeic acid, gallic acid, etc) are more reflexive than phenolic substances that cannot form quinones and therefore proposed that semi-quinones formation may respond with free thiol groups and amino acid side chains on the enzyme. This observation could be rationalized in terms of the effect of DMSO, because, in the present study, the pigments are dissolved in it. However, the sea urchin may have several bioactive compounds including phenol, flavonoid, carotenoid, omega 3 fatty acid, enzymes, astaxanthin, protein, and mineral, and so on, which can neutralize the DMSO effects (Sibiya et al., 2021; Cirino et al., 2017).

### 3.4 Antimicrobial activity

The inhibiting microbial growth data showed that both pigment extracts from shell and spine displayed low antibacterial effect against bacteria especially, *Bacillus subtilis* and *Bacillus pumilus* (MIC ranging from ≥1000 μg/ml) and no antibacterial activity against other tested bacterial strains. Whereas, previous studies have confirmed that extracts of echinochrome A and spinochrome C of various species of tropical sea urchin were efficient in destroying the strain of *Pseudomonas* strain, *E.coli*, *B. subtilis, Shewanella oneidensis*, and marine bacteria, *Vibrio* sp. and *Photobacterium* sp (Coates et al., 2018; Brasseur et al., 2017). The metal (e.g., Fe ^3+^) chelating properties, hydrophobicity, and free ortho-hydroxyl groups, and ketol structure of echinochrome pigments would harm microbial growth, some intracellular effects, and its potent antioxidant ability to scavenge oxidative/nitrosative radicals (Coates et al., 2018). According to the current results, it seems that some species-specific characteristics and differences in compositions of the sea urchins quinonoid pigments have been effective in antimicrobial activity (Vasileva et al., 2021). The seasonal change in algal/detritus diets and/or a variety of drift items based on the growth rates in urchins could be affected their microbial pigments. In contrast, previous work on Persian Gulf marine macroalgae as urchin phyto-diet showed considerable antimicrobial activity (Soleimani et al., 2018; Mashjoor et al., 2016).

### 3.5 Cytotoxicity and embryotoxicity

**Figure 6:**
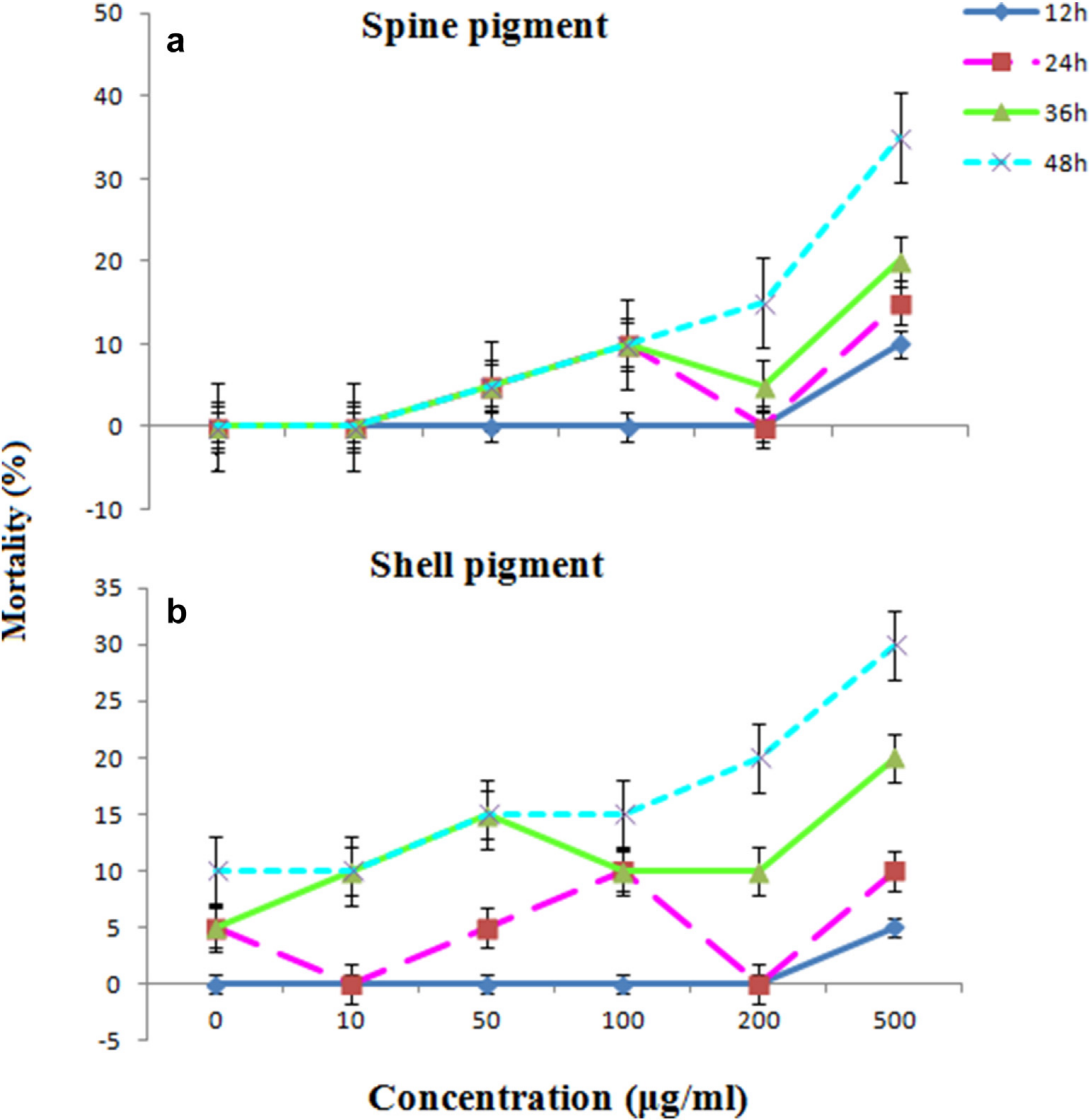
Mortality rate (%) of zebrafish embryos exposed to different concentration of the PHNQs pigment extracts from the *E.matheai* shell and spine over 48 h (A, B). Error bars represent ± one standard deviation from the mean of three replicates.

Table 3 and Figure 6 show cytotoxic brine shrimp activity and acute toxicity to zebrafish embryos for various concentrations of E.matheai shell and spine pigment extracts. At all concentrations, all pigment extracts had a <50 % mortality rate in brine shrimp (LC_50_ > 1000 μg/ml). As shown in Figure 6 A and B, 10–100 μg/mL of pigment extracts exhibited no toxicity to *D. rer*io embryos. Both 200 and 500 μg/mL of shell and spine pigments demonstrated low toxicity in zebrafish embryos, killing ∼30% (EC_50_ = 990 μg/mL) and ∼37% (EC_50_ = 708 μg/mL), respectively, at the end of the exposure (48 h). The results showed lower cytotoxic activity for the pigments extracts from *E. mathaei*. Given this, urchin algal species diets from the Qeshm Island shallow subtidal exhibited potent shrimp toxicity and cytotoxicity against MCF7, HeLa, and Vero (IC_50_ < 100 μg/ml) (Soleimani et al., 2018; Mashjoor et al., 2016). In agreement with our findings, Janeczko et al. (2018) displayed that 1,4-Naphthoquinone derivatives at low dosages (0.8–31.2 mg/L), show no toxicity toward zebrafish embryos. However, previous studies have shown that naphthoquinones and their acetyl-o-glucosides could act as cytotoxic agents through unrelated tubulin mechanisms (Sabutskia et al., 2017). Naphthoquinones have also been suggested to act as harmful or very toxic compounds against zebrafish (Song et al., 2010). In view of the mechanism of toxicity and according to the structure-activity relationship analysis, the interaction between the naphthoquinones and the target is likely mediated by 1-carbonyl and the hydrophobic fraction substituted in α-position of naphthoquinone via hydrogen bonding and hydrophobic interactions (Song et al., 2010).

**Table 3:** Cytotoxicity effect (% mortality) of the PHNQs pigment extracts from the shell and spine of sea urchin *E.matheai*.

Samples	∗Concentration (μg/ml)				LC_50_
	125	250	500	1000	
Shell pigment	2.05 ± 1.05	5.07 ± 1.09	7.5 ± 2.02	12.07 ± 3.32	LC_50_ > 1000
Spine pigment	3.73 ± 1.22	27.15 ± 5.02	31.29 ± 7.09	33.82 ± 9.12	LC_50_ > 1000

∗ Mean value ± SD (n = 3), P < 0.05.

## 4 Conclusions

The current study found that PHNQ pigments extracted from black type sea urchin *E. mathaei* shells and spines contain spinochrome A, B, and C, as well as echinochrome A, during the summer season. The pigments have also been shown to have antioxidant, anti-diabetic, anti-inflammatory, and antibacterial and cytotoxic activity. It is possible that dietary intake of natural phyto-biodynamic compounds contributed to the observed short-term increase in the bioactivity of sea urchin pigments. In conclusion, black type sea urchin *E. mathaei* shells and spines emerged as potent source for bioactive pigments of natural origin that can be used for medicinal applications.

## Declarations

### Author contribution statement

Soolmaz Soleimani: Performed the experiments; Analyzed and interpreted the data; Wrote the paper.

Sakineh Mashjoor: Conceived and designed the experiments; Performed the experiments; Analyzed and interpreted the data; Wrote the paper.

Morteza Yousefzadi: Conceived and designed the experiments; Contributed reagents, materials, analysis tools or data.

Manish Kumar: Analyzed and interpreted the data; Wrote the paper.

### Funding statement

This research did not receive any specific grant from funding agencies in the public, commercial, or not-for-profit sectors and proceed further with the article.

### Data availability statement

Data included in article/supplementary material/referenced in article.

### Declaration of interests statement

The authors declare no conflict of interest.

### Additional information

No additional information is available for this paper.
